# Supplementary material for: ProTargetMiner as a proteome signature library of anticancer molecules for functional discovery
Source: Nat Commun. 2019 Dec 16;10:5715. doi: 10.1038/s41467-019-13582-8 (PMC6915695; doi:10.1038/s41467-019-13582-8)
Supplement: Supplementary file 1 — Supplementary Information [file 41467_2019_13582_MOESM1_ESM.pdf]

SUPPLEMENTARY INFORMATION

ProTargetMiner as a proteome signature library of anticancer molecules for functional discovery

Saei et al.

Supplementary Figures

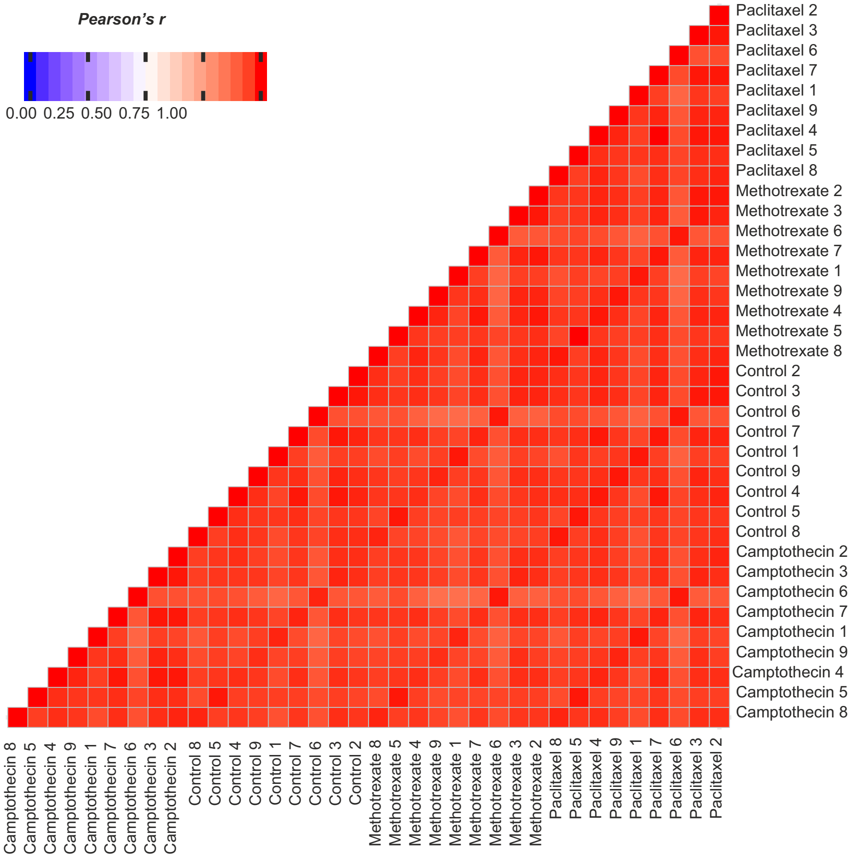

Supplementary Fig. 1 Reproducibility of proteomics measurements in the original ProTargetMiner dataset. Pearson correlation coefficient *r* was calculated for the control drugs present in all multiplexed experiments (no missing values were allowed). Experiments 1-9 are indicated with numbers. Source data are provided as a Source Data file.

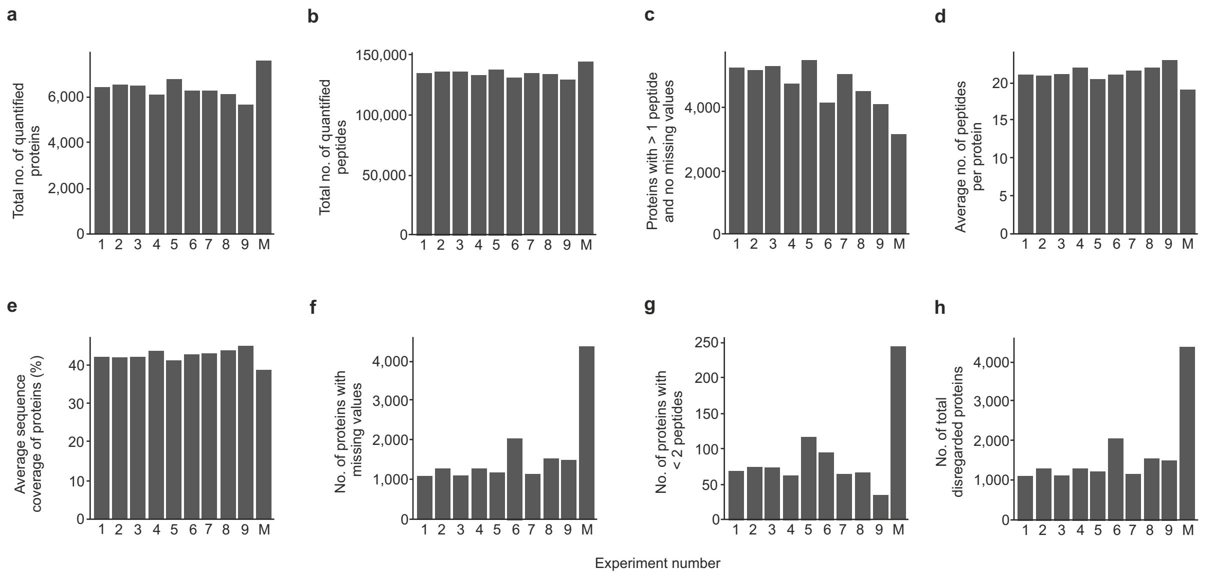

Supplementary Fig. 2 The comparison of the depth and quality of the original dataset experiments. a, total number of quantified proteins in each experiment 1-9 and merged original dataset M; **b,** total number of quantified peptides; **c,** proteins considered of high quality quantified with at least 2 peptides and no missing values in any of the replicates; **d,** average number of peptides per protein; **e,** average sequence coverage of proteins (%); **f,** number of proteins with missing values; **g,** number of proteins quantified with less than 2 peptides; **h,** number of proteins with less than 2 peptides and/or missing values. Source data are provided as a Source Data file.

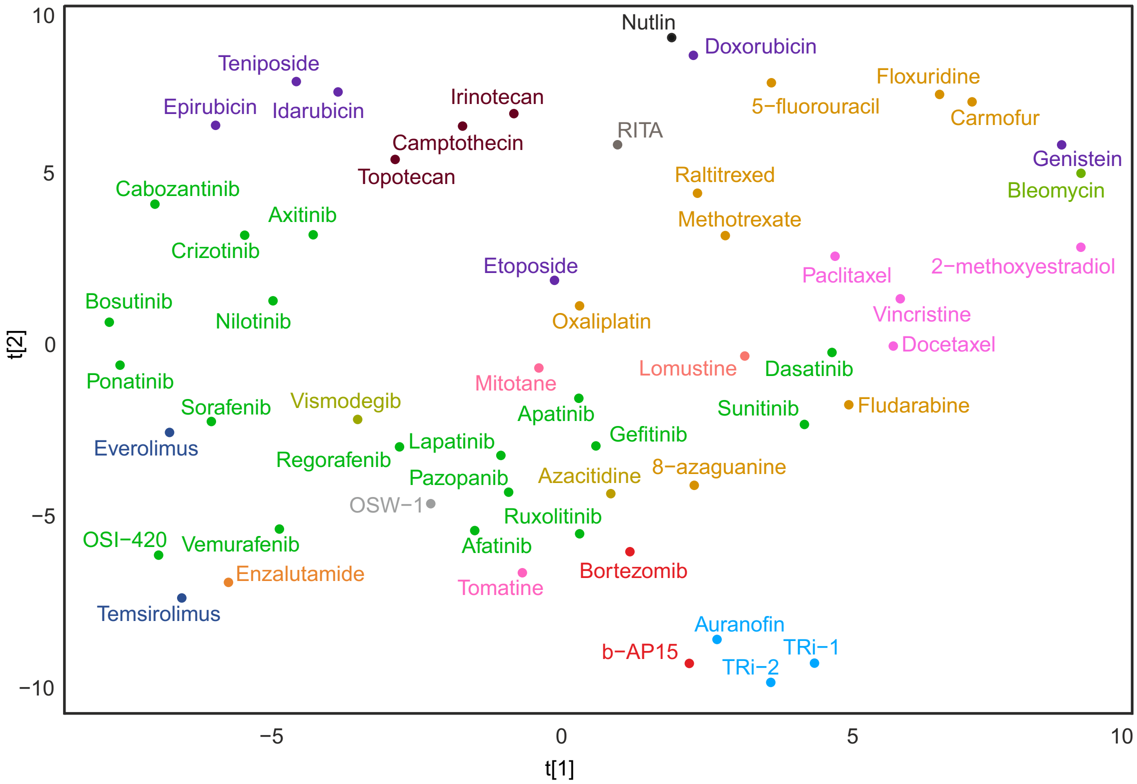

Supplementary Fig. 3 t-SNE analysis of proteome signatures. Molecules with similar mechanisms induce similar proteome changes and are found proximate on the t-SNE plot (“Death map”). Compounds in the same class (n=19) are differentiated with color. Figure made from Supplementary Data 1.

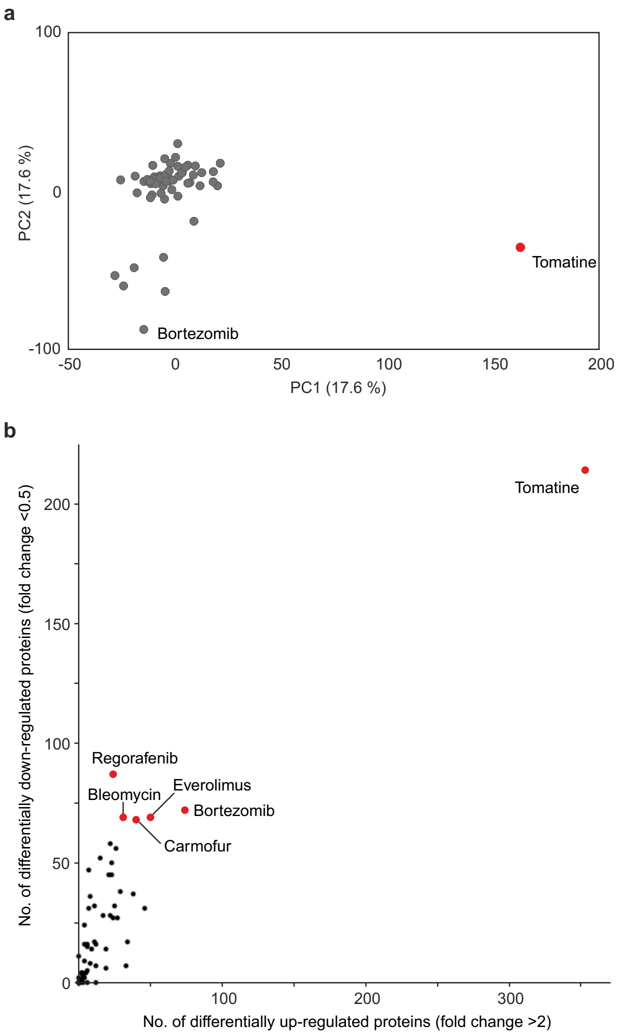

Supplementary Fig. 4 Tomatine has a peculiar effect on the cell proteome. a, tomatine as a big outlier in PCA. **b,** the number of differentially regulated proteins (fold change vs. control >2 and < 0.5) for tomatine compared to other drugs (among 3,421 proteins with no missing values). For panel a, the raw data from ProteomeXchange dataset PXD009775 with no missing values was used. Source data are provided as a Source Data file.

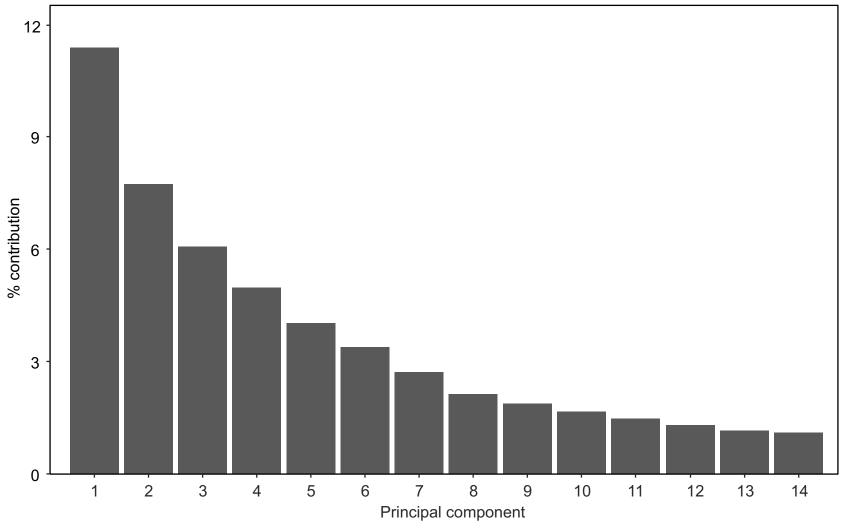

Supplementary Fig. 5 The contribution of each PCA component to the separation of proteome signatures. Source data are provided as a Source Data file.

a

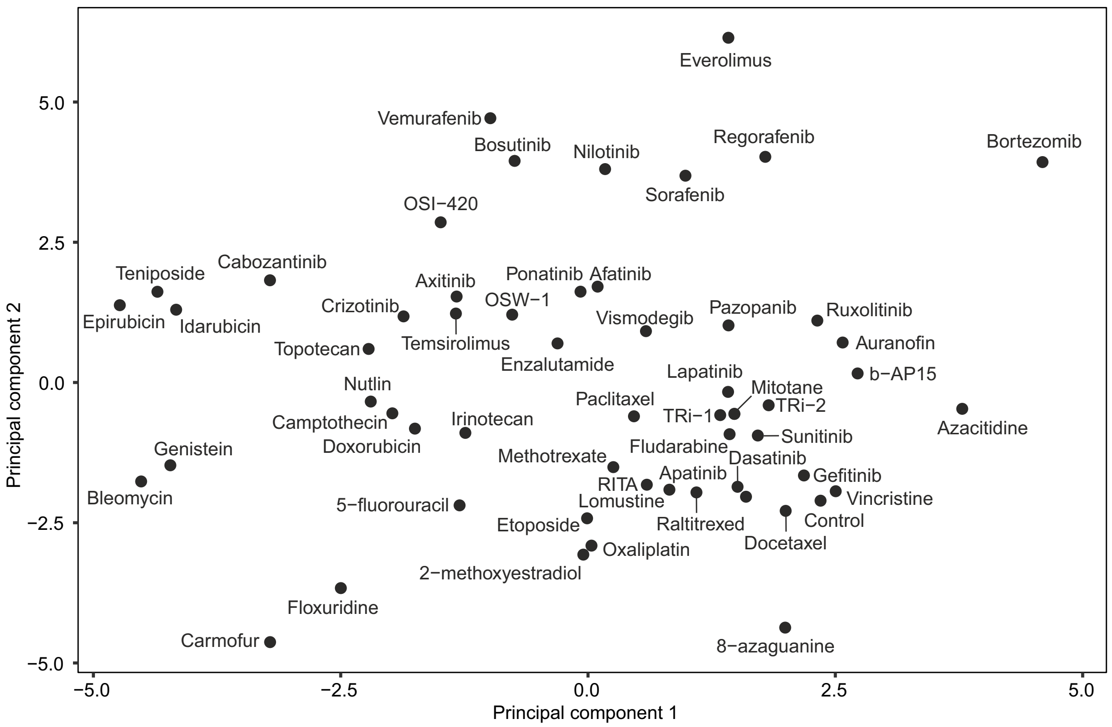

b

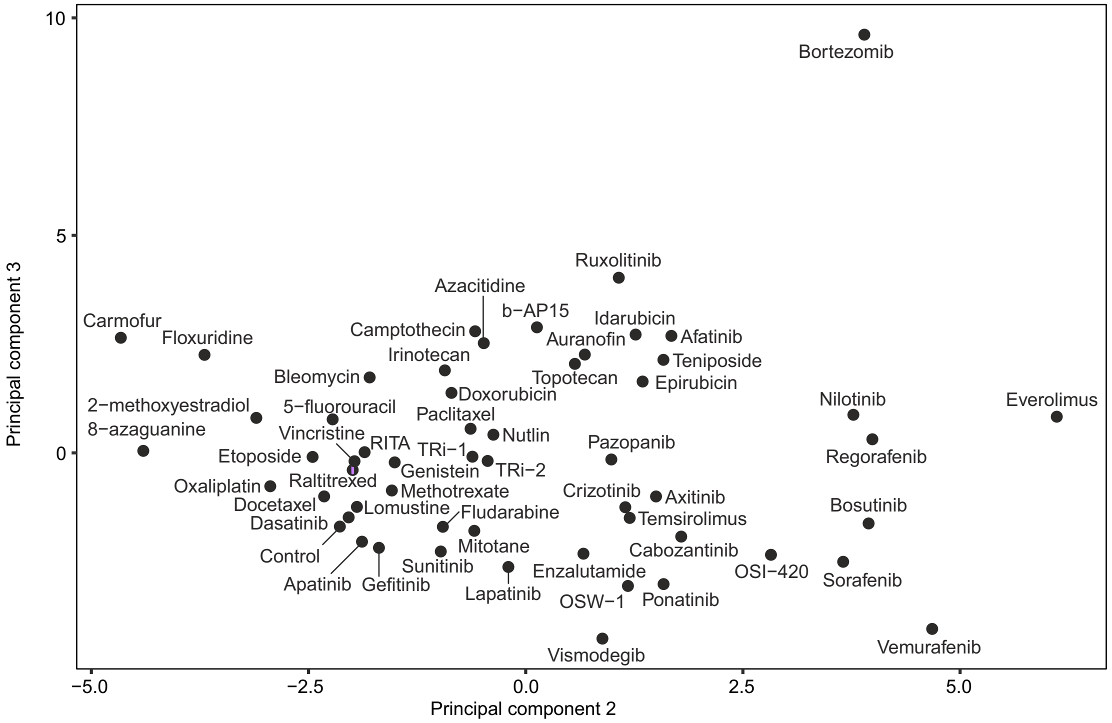

Supplementary Fig. 6 PCA of proteome signatures. The first 3 components are shown: **a,** component 1 vs. 2 and **b,** component 2 vs. 3. Source data are provided as a Source Data file.

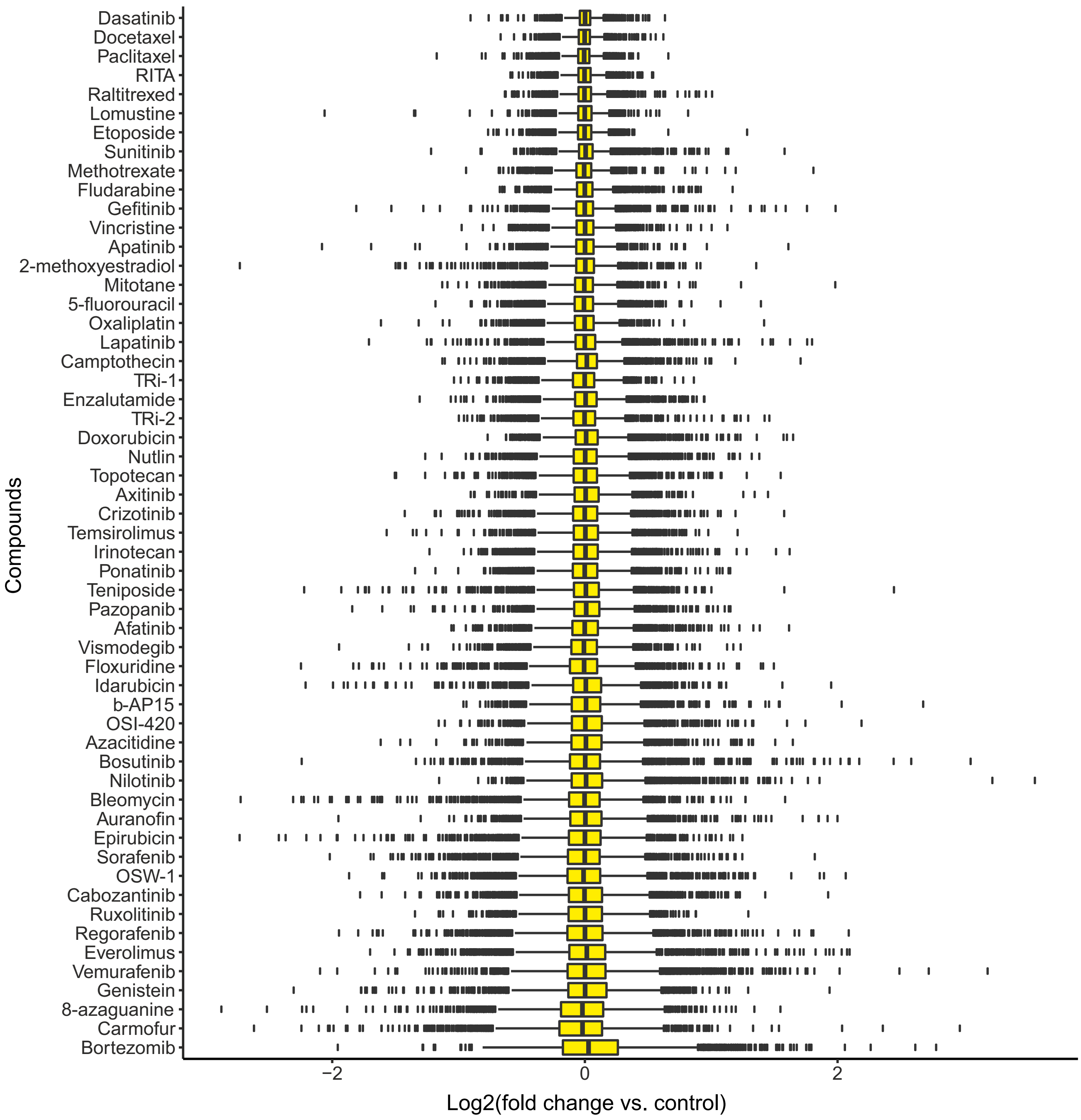

Supplementary Fig. 7 The degree of drug-induced proteome changes. Total variation of proteome changes might be indicative of compound specificity. Source data are provided as a Source Data file. Center line, median; box limits contain 50%; upper and lower quartiles, 75% and 25%; maximum, greatest value excluding outliers; minimum, least value excluding outliers; outliers, more than 1.5 times of upper and lower quartiles. Supplementary Data 1 was used for generation of figure.

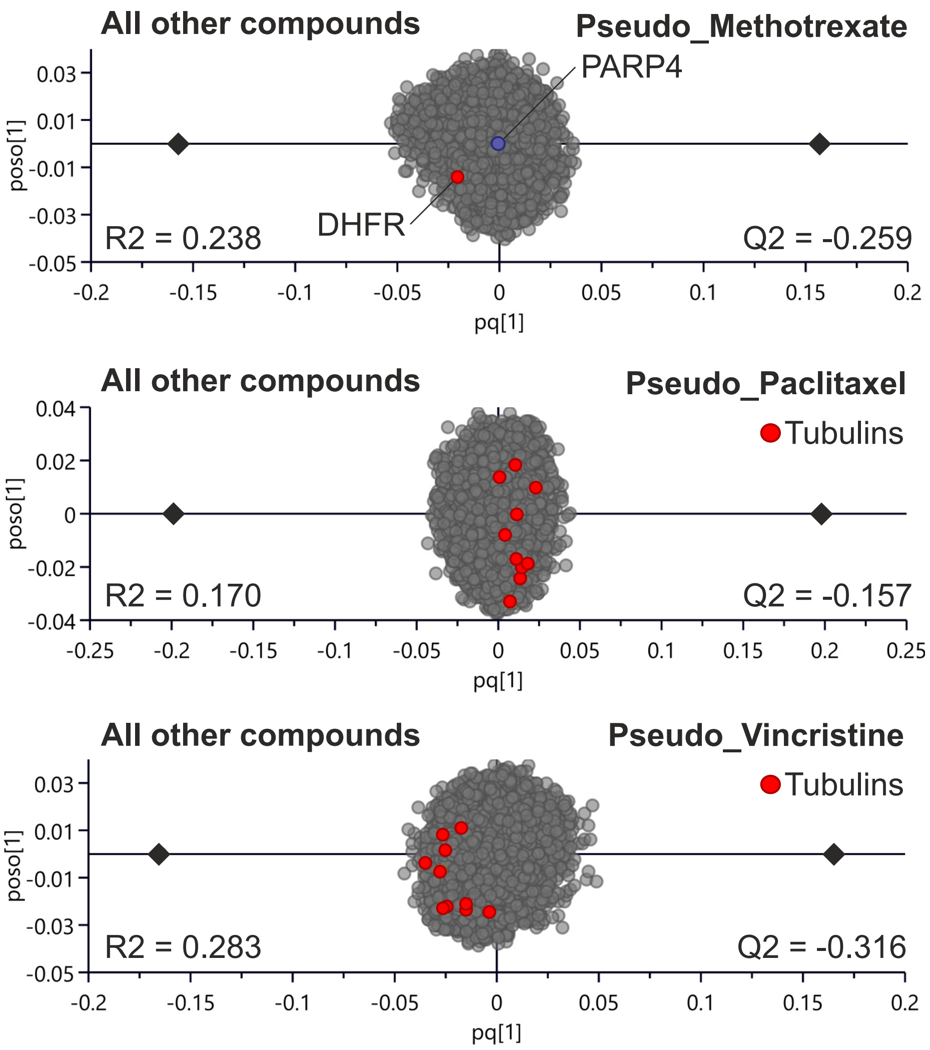

Supplementary Fig. 8 Cross-validation of OPLS-DA models. For OPLS-DA models in **Fig. 4** for methotrexate, paclitaxel and vincristine, pseudo models were built. First, the data for these compounds were removed one by one from the dataset and then, 3 random columns were selected for building the pseudo models against the rest of the dataset. The proteins shown in red circles are the specific proteins that were also highlighted in **Fig. 4**. Supplementary Data 1 was used for generation of figure.

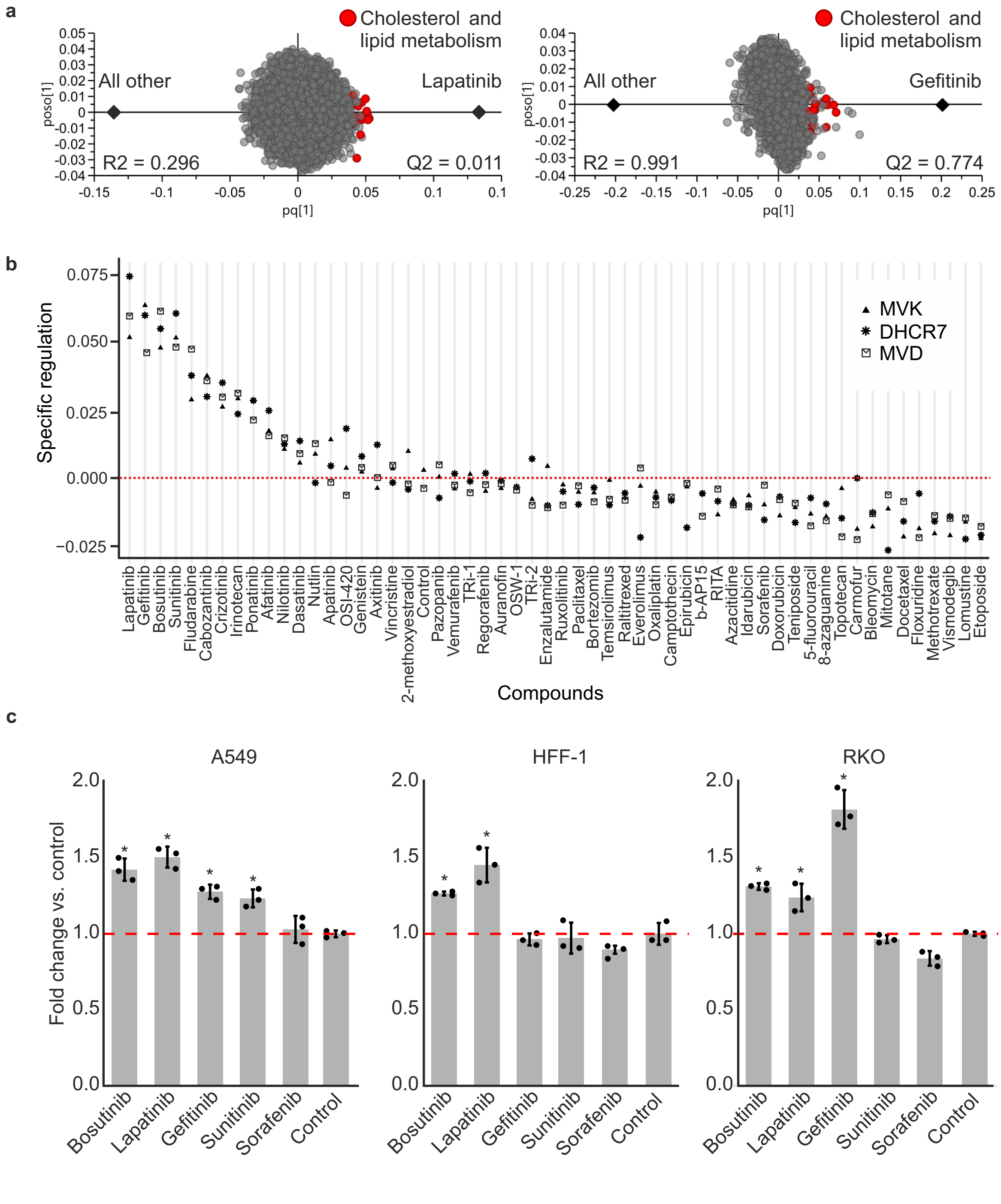

Supplementary Fig. 9 ProTargetMiner yields mechanistically relevant molecular information for kinase inhibitors. a, up-regulation of proteins involved in chole(sterol) and lipid metabolism in response to lapatinib and gefitinib as representative examples (the pathway proteins are shown in red circles). **b,** exemplary proteins involved in cholesterol metabolism specifically

up-regulated in response to kinase inhibitors vs. other compounds. **c,** the effect of selected kinase inhibitors on cellular total cholesterol levels. Cellular total cholesterol was measured upon treatment with 4 μM of the compounds over a 20 h treatment period. Sorafenib was used as a negative control. (*$p < 0.05$, two-sided Student t-test, n=3 biologically independent experiments). Data are represented as mean±s.d. Supplementary Data 1 was used for generation of panel a. Supplementary Data 3 was used for generation of panel b. Source data are provided as a Source Data file.

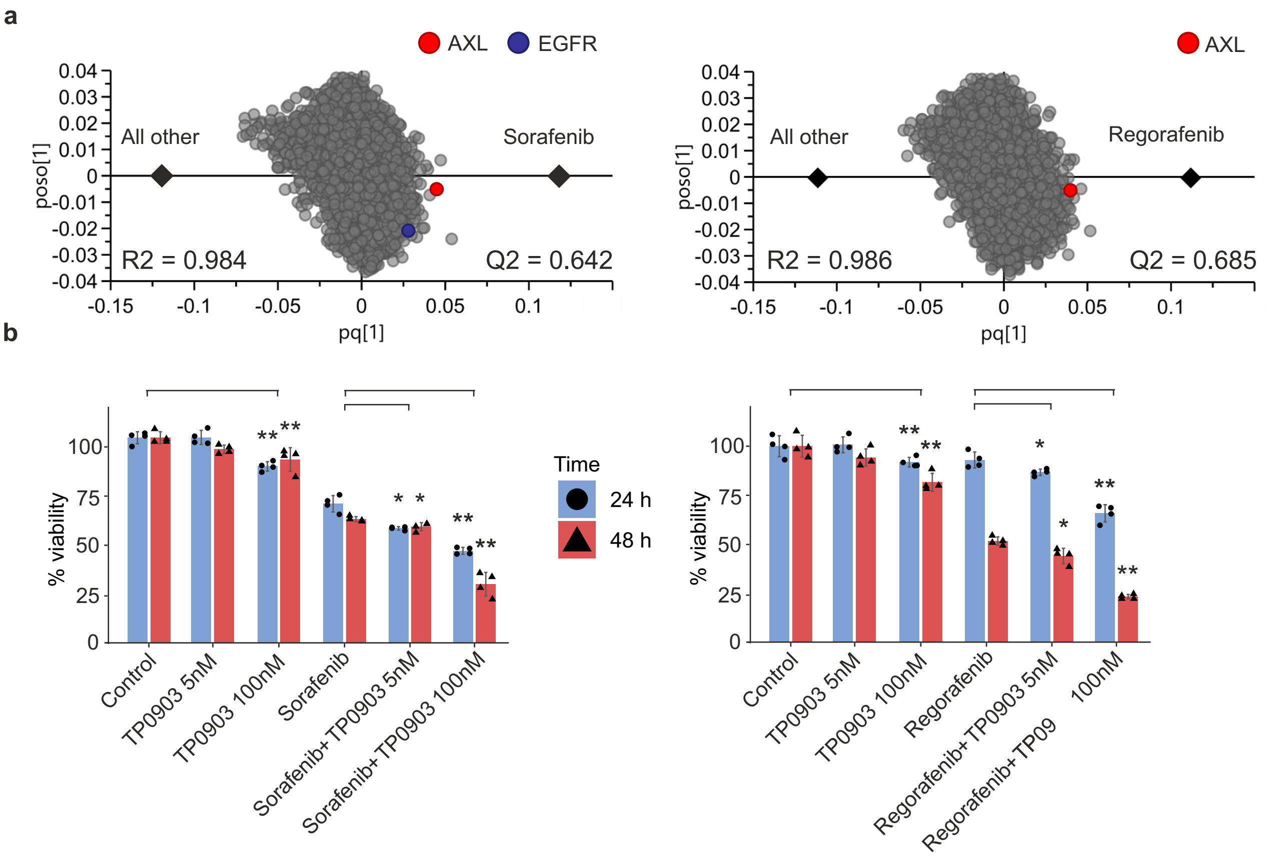

Supplementary Fig. 10 ProTargetMiner can reveal potential resistance factors. a, models contrasting sorafenib and regorafenib against 54 other compounds, showing the specific up-regulation of AXL (red circles) upon treatment of A549 cells (note the up-regulation of EGFR by sorafenib (blue circle)). **b,** treatment of cells with non-cytotoxic concentrations of TP0903, a specific and nanomolar AXL inhibitor, sensitized A549 cells to sorafenib and regorafenib in 24 and 48h (*p < 0.05, **p < 0.005, two-sided Student t-test, n=4 biologically independent experiments). Data are represented as mean±s.d. Supplementary Data 1 was used for generation of panel a. Source data are provided as a Source Data file.

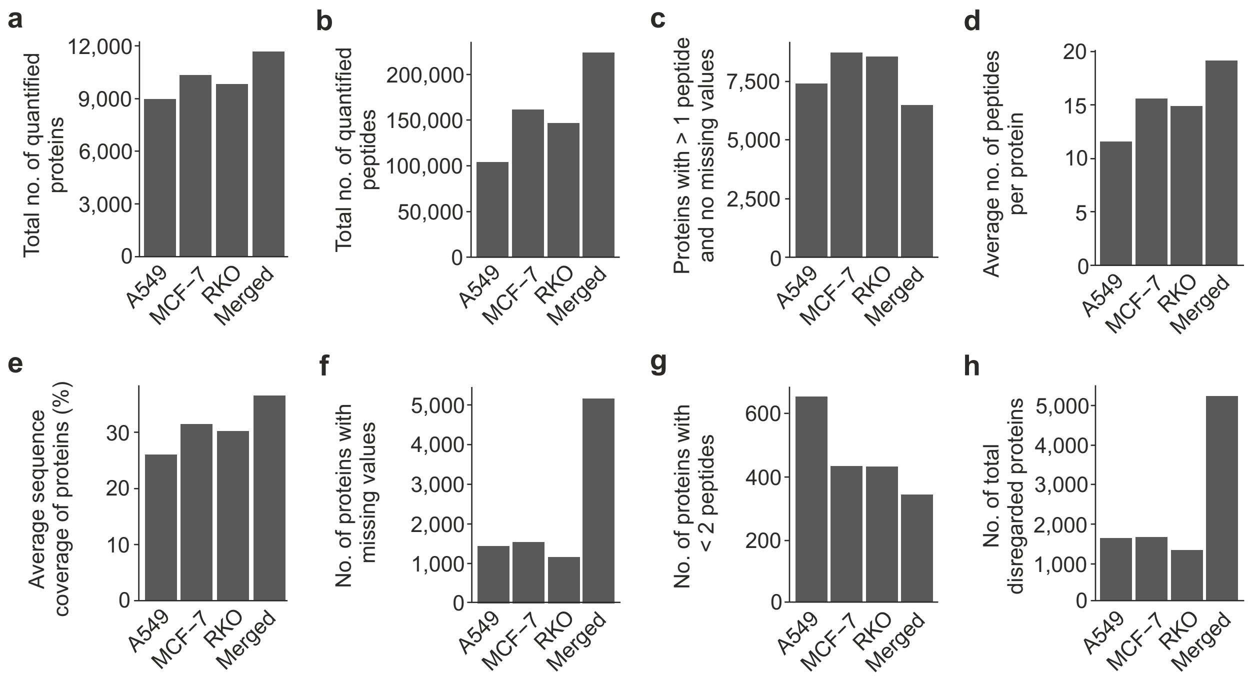

Supplementary Fig. 11 The comparison of the depth and quality of deep datasets (also compared with the merged deep dataset). a, total number of quantified proteins; **b,** total number of quantified peptides; **c,** proteins considered of high quality quantified with at least 2 peptides and no missing values in any of the replicates; **d,** average number of peptides per protein; **e,** average sequence coverage of proteins (%); **f,** number of proteins with missing values; **g,** number of proteins quantified with less than 2 peptides; **h,** number of proteins with less than 2 peptides and/or missing values. Source data are provided as a Source Data file.

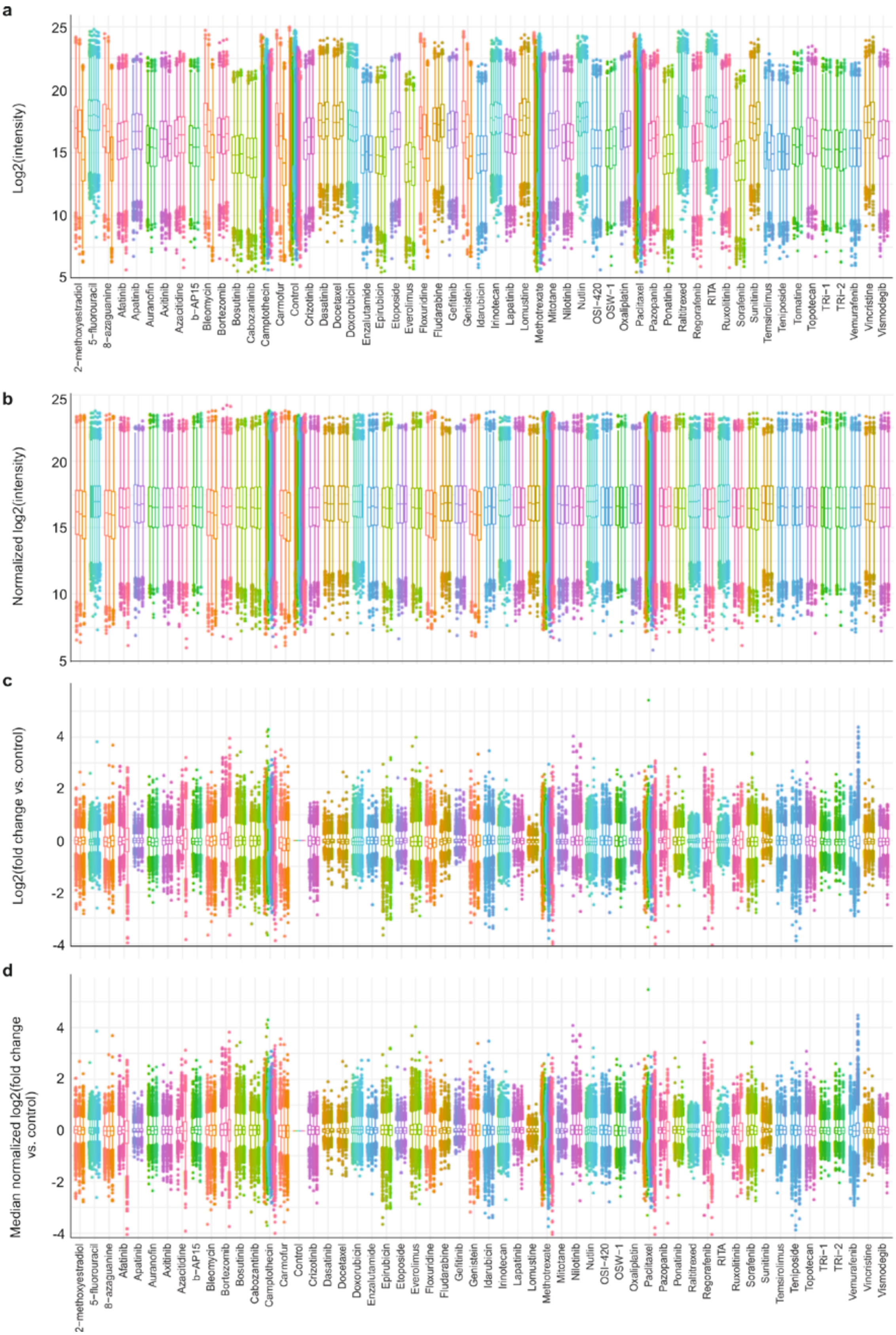
a
Log2(intensity)
25
20
15
10
5
2−methoxyestradiol
5−fluorouracil
8−azaguanine
Afatinib
Apatinib
Auranofin
Axitinib
Azacitidine
b−AP15
Bleomycin
Bortezomib
Bosutinib
Cabozantinib
Camptothecin
Carmofur
Control
Crizotinib
Dasatinib
Docetaxel
Doxorubicin
Enzalutamide
Epirubicin
Etoposide
Everolimus
Floxuridine
Fludarabine
Gefitinib
Genistein
Idarubicin
Irinotecan
Lapatinib
Lomustine
Methotrexate
Mitotane
Nilotinib
Nutlin
OSI−420
OSW−1
Oxaliplatin
Paclitaxel
Pazopanib
Ponatinib
Raltitrexed
Regorafenib
RITA
Ruxolitinib
Sorafenib
Sunitinib
Temsirolimus
Teniposide
Tomatine
Topotecan
TRi−1
TRi−2
Vemurafenib
Vincristine
Vismodegib
b
Normalized log2(intensity)
25
20
15
10
5
c
Log2(fold change vs. control)
4
2
0
-2
-4
d
Median normalized log2(fold change vs. control)
4
2
0
-2
-4
2−methoxyestradiol
5−fluorouracil
8−azaguanine
Afatinib
Apatinib
Auranofin
Axitinib
Azacitidine
b−AP15
Bleomycin
Bortezomib
Bosutinib
Cabozantinib
Camptothecin
Carmofur
Control
Crizotinib
Dasatinib
Docetaxel
Doxorubicin
Enzalutamide
Epirubicin
Etoposide
Everolimus
Floxuridine
Fludarabine
Gefitinib
Genistein
Idarubicin
Irinotecan
Lapatinib
Lomustine
Methotrexate
Mitotane
Nilotinib
Nutlin
OSI−420
OSW−1
Oxaliplatin
Paclitaxel
Pazopanib
Ponatinib
Raltitrexed
Regorafenib
RITA
Ruxolitinib
Sorafenib
Sunitinib
Temsirolimus
Teniposide
Topotecan
TRi−1
TRi−2
Vemurafenib
Vincristine
Vismodegib

Supplementary Fig. 12 The effect of normalization on data distribution and median stabilization. The data distribution before (**a**) and after (**b**) median intensity normalization for all the replicates. The data distribution before (**c**) and after (**d**) median fold change normalization. Note the stabilization of the median intensity and median fold changes in each step (Center line, median; box limits contain 50%; upper and lower quartiles, 75% and 25%; maximum, greatest value excluding outliers; minimum, least value excluding outliers; outliers, more than 1.5 times of upper and lower quartiles). Data for each drug is shown in a different color. Supplementary Data 1 as well as raw data from ProteomeXchange dataset PXD009775 (original ProTargetMiner data) was used for generation of figure.

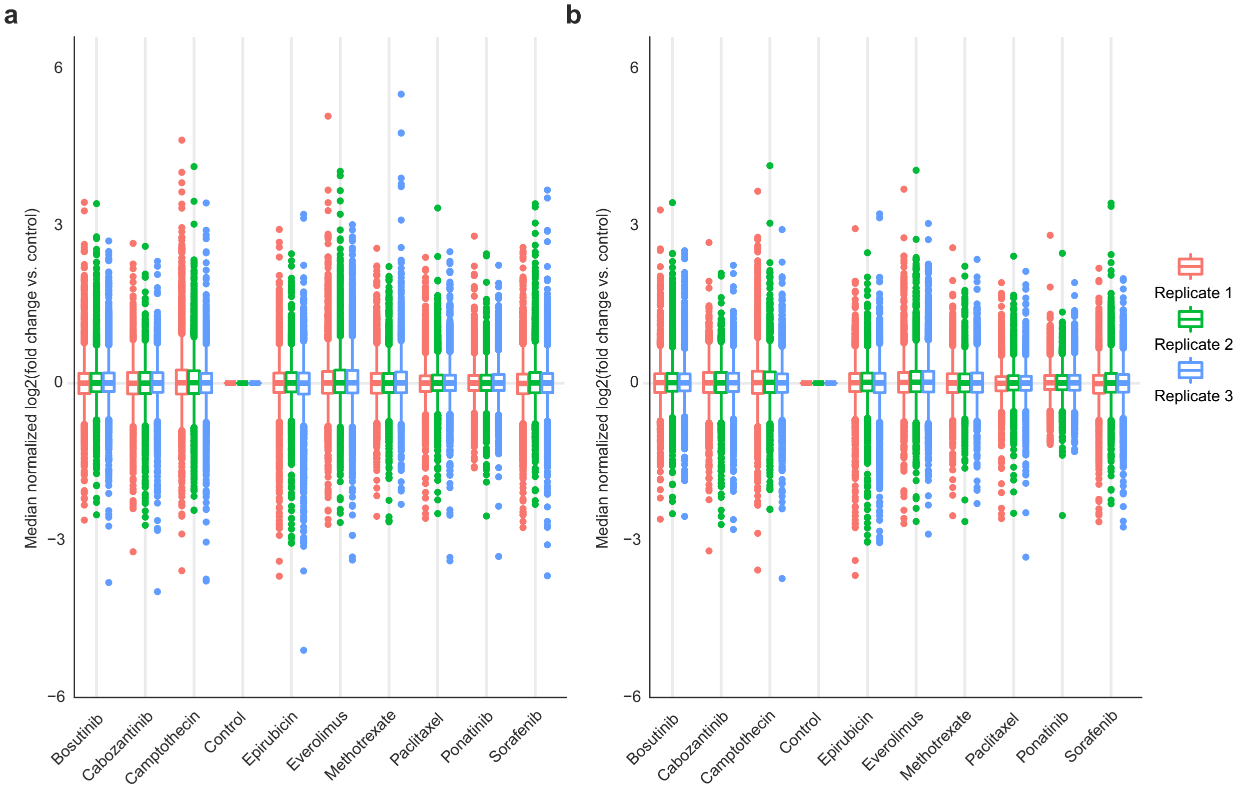

Supplementary Fig. 13 Median fold change normalization of data from experiment 4 in the original dataset. The distribution of relative protein abundances from experiment 4, comparing the whole dataset normalization (**a**) with normalization of only experiment 4 (**b**) (Center line, median; box limits contain 50%; upper and lower quartiles, 75% and 25%; maximum, greatest value excluding outliers; minimum, least value excluding outliers; outliers, more than 1.5 times of upper and lower quartiles). Data from each replicate is shown in a different color. Supplementary Data 1 as well as raw data from ProteomeXchange dataset PXD009775 (original ProTargetMiner data) was used for generation of figure.

Supplementary Tables

Supplementary Table 1. Compounds, curated targets from DrugBank (November 2019), class assignments and LC50s (μM)

Drug name	Known Targets	Class	LC50 (A549)	LC50 (MCF-7)	LC50 (RKO)
Axitinib	FLT1, FLT4, KDR	Kinase inhibitor	50	25	50
Lapatinib	ERBB2, EGFR	Kinase inhibitor	20	8	8
Crizotinib	ALK, MET	Kinase inhibitor	3	3	10
Regorafinib	FLT1, KDR, FLT4, KIT, PDGFRA, PDGFRB, FGFR1, FGFR2, DDR2, TEK, NTRK1, RAF1, EPHA2, MAPK11, BRAF, FRK, ABL1, RET	Kinase inhibitor	15	7.5	6
Ruxolitinib	JAK1, JAK2	Kinase inhibitor	30	37.5	20
Afatinib	ERBB2, ERBB4, EGFR	Kinase inhibitor	7	3	5
Cabozantinib	MET, RET, KDR	kinase inhibitor	7.5	50	25
Ponatinib	BCR, ABL1, KIT, RET, TEK, FLT3, FGFR1, FGFR2, FGFR3, FGFR4, LCK, SRC, LYN, KDR, PDGFRA	kinase inhibitor	3	3	2
Sorafenib	RAF1, BRAF, FLT4, KDR, FLT3, PDGFRB, KIT, FGFR1, RET, FLT1,	kinase inhibitor	10	20	7.5
Bosutinib	LYN, BCR, ABL1, HCK, SRC, CDK2, MAP2K1, MAP3K2, MAP2K2, CAMK2G	kinase inhibitor	7	15	10
Sunitinib	PDGFRB, FLT1, KIT, KDR, CSF1R, FLT4, FLT3, PDGFRA	kinase inhibitor	7.5	7.5	10
Dasatinib	ABL1, SRC, EPHA2, LCK, YES1, KIT, PDGFRB, STAT5B, ABL2, FYN, BTK, NR4A3, BCR, CSK, EPHA5, EPHB4, FGR, FRK, HSPA8, LYN, ZAK, MAPK14, PPAT	kinase inhibitor	25	25	15
Apatinib	KDR (VEGFR2)	kinase inhibitor	25	40	25
Gefitinib	EGFR	kinase inhibitor	20	30	37.5
Vemurafenib	BRAF	kinase inhibitor	35	40	25
OSI-420	EGFR	kinase inhibitor	20	25	30
Nilotinib	KIT, ABL1	Kinase inhibitor	20	4	10
Pazopanib	FLT1, KDR, PDGFRA, PDGFRB, FLT4, FGFR3, KIT, FGF1, ITK, SH2B3	Kinase inhibitor	10	50	17.5
Vismodegib	SMO	Hedgehog signaling pathway	50	40	50
Azacitidine	DNMT1, nucleotide	DNA methyltransferase inhibitor	10	50	10
Everolimus	mTOR	mTOR inhibitor	50	50	35
Temsirolimus	mTOR	mTOR inhibitor	25	25	8
Fludarabine	POLA1, RRM1, DCK, nucleotide	Antimetabolite	25	50	25
Oxaliplatin	Nucleotide	Antimetabolite	25	50	25

5-fluorouracil	TYMS, Nucleotide	Antimetabolite	50	50	25
Methotrexate	DHFR	Antimetabolite	1	50	0.025
Raltitrexed	FPGS, TYMS	Antimetabolite	50	-	-
Carmofur	TYMS and AC (acid ceramidase) inhibitor	Antimetabolite	50	50	50
Floxuridine	TYMS	Antimetabolite	25	50	25
8-azaguanine	PNP	Antimetabolite	35	50	30
Vincristine	TUBB, TUBA4A	Tubulin polymerization inhibitors	1	0.005	50
2-methoxyestradiol	HIF1A, COMT, CYP1A1, CYP1B1, CYP19A1	Tubulin polymerization inhibitors	3.5	5	0.5
Paclitaxel	BCL2, TUBB1, NR1I2, MAP4, MAP2, MAPT	Tubulin depolymerization inhibitors	0.05	0.05	0.01
Docetaxel	TUBB1, bcl2, MAP2, MAP4, MAPT, NR1I2	Tubulin depolymerization inhibitors	0.5	0.4	1
Genistein	ESR2, ESR1, TOP2A, PTK2B, NCOA2, NCOA1	Topoisomerase II inhibitor, protein kinase inhibitor	50	50	50
Epirubicin	CHD1, TOP2A, nucleotide	Topoisomerase II inhibitor, DNA intercalation	1	0.75	0.3
Doxorubicin	TOP2A, Nucleotide	Topoisomerase II inhibitor, DNA alkylating	0.75	0.75	0.05
Etoposide	TOP2A, TOP2B	Topoisomerase II inhibitor	10	50	50
Idarubicin	TOP2A, Nucleotide	Topoisomerase II inhibitor, DNA alkylating	0.4	0.75	0.04
Lomustine	STMN4, nucleotide	Alkylating agent	50	50	50
Teniposide	TOP2A	Topoisomerase II inhibitor	4	35	1
Topotecan	TOP1MT, TOP1, nucleotide	Topoisomerase I inhibitor	0.4	5	5
Irinotecan	TOP1, TOP1MT	Topoisomerase I inhibitor	50	50	17.5
Camptothecin	TOP1	Topoisomerase I inhibitor	3	1	5
Mitotane	CYP11B1, FDX1	Unknown, peripheral metabolism of steroids and adrenocortical steroid inhibitor	50	50	50
RITA	p53	p53 activator	10	50	50
Nutlin	MDM2-p53 interaction	MDM2 inhibitor	25	37.5	40
Enzalutamide	AR (androgen receptor)	Androgen receptor inhibitor	35	25	20
Bleomycin	LIG1, LIG3, nucleotide	Induction of DNA strand breaks	50	50	5
Bortezomib	PSMB5, PSMB1	Proteasome inhibitor	0.15	50	40
b-AP15	UCHL5, USP14	Proteasome deubiquitinase inhibitor	3	-	-
Auranofin	TrxR1, PRDX5, IKBKB, (USP14 and UCHL5 inhibition has also been seen)	Thioredoxin reductase 1 inhibitor, proteasome deubiquitinase inhibitor	6.5	-	-
TRi-1	TrxR1	Thioredoxin reductase 1 inhibitor	30	-	-
TRi-2	TrxR1	Thioredoxin reductase 1 inhibitor	17.5	-	-
Tomatine	Acetylcholinesterase, membrane disruption	Unknown, disruption of membrane, potential inhibition of the proteasome	3	-	-
OSW-1	OSBP1	Unknown, OSBP binder	0.5	-	-

Supplementary Table 2. TMT-10 multiplexing information for the ProTargetMiner experiments.

TMT tag	Experiment 1	Experiment 2	Experiment 3	Experiment 4	Experiment 5	Experiment 6	Experiment 7	Experiment 8	Experiment 9	All three deep experiments
**126**	5-fluorouracil	Regorafenib	Vismodegib	Cabozantinib	Fludarabine	-	Idarubicin	Carmofur	OSW-1	Control
**127N**	Methotrexate	Methotrexate	Methotrexate	Methotrexate	Methotrexate	Methotrexate	Methotrexate	Methotrexate	Methotrexate	8-azaguanine
**127C**	Paclitaxel	Paclitaxel	Paclitaxel	Paclitaxel	Paclitaxel	Paclitaxel	Paclitaxel	Paclitaxel	Paclitaxel	Raltitrexed
**128N**	Doxorubicin	Afatinib	Axitinib	Ponatinib	Sunitinib	Etoposide	Temsirolimus	Genistein	Auranofin	Topotecan
**128C**	Irinotecan	Bortezomib	Nilotinib	Everolimus	Dasatinib	Gefitinib	Enzalutamide	Bleomycin	TRi-1	Floxuridine
**129N**	Camptothecin	Camptothecin	Camptothecin	Camptothecin	Camptothecin	Camptothecin	Camptothecin	Camptothecin	Camptothecin	Nutlin
**129C**	RITA	Ruxolitinib	Crizotinib	Sorafenib	Docetaxel	Oxaliplatin	Vemuratenib	8-Azaguanine	TRi-2	Dasatinib
**130N**	Nutlin	Azacitidine	Topotecan	Bosutinib	Lomustine	Mitotane	OSI-420	Floxuridine	b-AP15	Gefitinib
**130C**	Raltitrexed	Pazopanib	Lapatinib	Epirubicin	Vincristine	Apatinib	Teniposide	2-methoxyestradiol	Tomatine	Vincristine
**131**	Control	Control	Control	Control	Control	Control	Control	Control	Control	Bortezomib
