# Supplementary material for: ProTargetMiner as a proteome signature library of anticancer molecules for functional discovery
Source: Nat Commun. 2019 Dec 16;10:5715. doi: 10.1038/s41467-019-13582-8 (PMC6915695; doi:10.1038/s41467-019-13582-8)
Supplement: Supplementary file 3 — Description of Additional Supplementary Files [file 41467_2019_13582_MOESM3_ESM.docx]

**Description of Supplementary Files**

**File Name:** Supplementary Data 1

**Description:** The original ProTargetMiner dataset with 55 drugs (log2 normalized ratios, excluding tomatine).

**File Name:** Supplementary Data 2

**Description:** Cluster compositions and the Gene Ontology (GO), Molecular Function (MF) and Cellular Component (CC) pathways enriched for the protein clusters in Fig. 2.

**File Name:** Supplementary Data 3

**Description:** The specificity of each protein in response to each compound against all other compounds in OPLS models.

**File Name:** Supplementary Data 4

**Description:** The deep proteomics dataset with 9 drugs in A549 cells.

**File Name:** Supplementary Data 5

**Description:** The deep proteomics dataset with 9 drugs in MCF-7 cells.

**File Name:** Supplementary Data 6

**Description:** The deep proteomics dataset with 9 drugs in RKO cells.

**File Name:** Supplementary Data 7

**Description:** The merged deep proteomics dataset with 9 drugs in A549, MCF-7 and RKO cells.
