# Supplementary material for: ProTargetMiner as a proteome signature library of anticancer molecules for functional discovery
Source: Nat Commun. 2019 Dec 16;10:5715. doi: 10.1038/s41467-019-13582-8 (PMC6915695; doi:10.1038/s41467-019-13582-8)
Supplement: Supplementary file 11 — Reporting Summary [file 41467_2019_13582_MOESM11_ESM.pdf]

nature research

Corresponding author(s): Roman A. Zubarev

Reporting Summary

Nature Research wishes to improve the reproducibility of the work that we publish. This form provides structure for consistency and transparency in reporting. For further information on Nature Research policies, see Authors & Referees and the Editorial Policy Checklist.

Statistical parameters

When statistical analyses are reported, confirm that the following items are present in the relevant location (e.g. figure legend, table legend, main text, or Methods section).

n/a	Confirmed	
	x	The exact sample size (*n*) for each experimental group/condition, given as a discrete number and unit of measurement
	x	An indication of whether measurements were taken from distinct samples or whether the same sample was measured repeatedly
	x	The statistical test(s) used AND whether they are one- or two-sided<br>*Only common tests should be described solely by name; describe more complex techniques in the Methods section.*
x		A description of all covariates tested
x		A description of any assumptions or corrections, such as tests of normality and adjustment for multiple comparisons
	x	A full description of the statistics including central tendency (e.g. means) or other basic estimates (e.g. regression coefficient) AND variation (e.g. standard deviation) or associated estimates of uncertainty (e.g. confidence intervals)
	x	For null hypothesis testing, the test statistic (e.g. *F*, *t*, *r*) with confidence intervals, effect sizes, degrees of freedom and *P* value noted<br>*Give P values as exact values whenever suitable.*
x		For Bayesian analysis, information on the choice of priors and Markov chain Monte Carlo settings
	x	For hierarchical and complex designs, identification of the appropriate level for tests and full reporting of outcomes
x		Estimates of effect sizes (e.g. Cohen's *d*, Pearson's *r*), indicating how they were calculated
	x	Clearly defined error bars<br>*State explicitly what error bars represent (e.g. SD, SE, CI)*

Our web collection on statistics for biologists may be useful.

Software and code

Policy information about availability of computer code

Data collection	Thermo Xcalibur 4.0
Data analysis	Excel 2013, MaxQuant 1.5.6.5, SIMCA 15, R project, STRING version 10.5 (http://string-db.org)

For manuscripts utilizing custom algorithms or software that are central to the research but not yet described in published literature, software must be made available to editors/reviewers upon request. We strongly encourage code deposition in a community repository (e.g. GitHub). See the Nature Research guidelines for submitting code & software for further information.

Data

Policy information about availability of data

All manuscripts must include a data availability statement. This statement should provide the following information, where applicable:

- Accession codes, unique identifiers, or web links for publicly available datasets
- A list of figures that have associated raw data
- A description of any restrictions on data availability

The LC-MS/MS raw data files are deposited in the jPOST repository of the ProteomeXchange Consortium under the dataset identifier PXD009775 (original ProTargetMiner data), PXD009644 (deep proteomics set for A549 cells) and PXD013134 (deep proteomics set for MCF-7 and RKO cells)). The extracted protein abundances data and relevant outputs of data analysis are provided in supplementary data cited in the text. All the figures are associated with the raw data.

April 2018

Field-specific reporting

Please select the best fit for your research. If you are not sure, read the appropriate sections before making your selection.

☒ Life sciences ☐ Behavioural & social sciences ☐ Ecological, evolutionary & environmental sciences

For a reference copy of the document with all sections, see nature.com/authors/policies/ReportingSummary-flat.pdf

Life sciences study design

All studies must disclose on these points even when the disclosure is negative.

Sample size	Biological triplicates (or in some cases more replicates) have been used in the study to gain appropriate power for discoveries.
Data exclusions	In total, 144,075 peptides attributed to 7,328 proteins were quantified in all experiments, with at least 2 unique peptides per protein. After selecting only proteins quantified for at least 50 drugs, the list was reduced to 4,557 proteins. All subsequent analyses were performed with the reduced dataset. In the deep proteomics validation dataset, only proteins with no missing values for all the treatments were kept and used.
Replication	All experiments were performed in independent biological triplicates as detailed in the paper. In all the nine proteomics experiments for the original ProTargetMiner dataset, methotrexate, paclitaxel and camptothecin were included (in triplicates) to serve as quality control. The first pilot proteomics experiment was performed in five replicates. The LC50 measurements were also performed in at least three biological replicates.
Randomization	Randomization was not required for proteomics samples in this work due to multiplexing.
Blinding	Blinding was not performed in this study.

Reporting for specific materials, systems and methods

Materials & experimental systems

n/a	Involved in the study
☒	☐ Unique biological materials
☒	☐ Antibodies
☐	☒ Eukaryotic cell lines
☒	☐ Palaeontology
☒	☐ Animals and other organisms
☒	☐ Human research participants

Methods

n/a	Involved in the study
☒	☐ ChIP-seq
☒	☐ Flow cytometry
☒	☐ MRI-based neuroimaging

Eukaryotic cell lines

Policy information about cell lines

Cell line source(s)	ATCC, USA
Authentication	Since passage number 2 cells were thawed and used from the ATCC source in all the experiments, no authentication was performed.
Mycoplasma contamination	Cells were routinely tested for mycoplasma by MycoAlert Mycoplasma Detection Kit (#Cat: 11650261, Thermo Fisher Scientific)
Commonly misidentified lines (See ICLAC register)	To the best of our knowledge no misidentified cell lines have been used in this study
